# Prediction of benign and malignant pulmonary nodules using preoperative CT features: using PNI-GARS as a predictor

**DOI:** 10.3389/fimmu.2024.1446511

**Published:** 2024-11-20

**Authors:** Yuxin Zhan, Feipeng Song, Wenjia Zhang, Tong Gong, Shuai Zhao, Fajin Lv

**Affiliations:** ^1^ School of Science, Chongqing University of Technology, Chongqing, China; ^2^ Department of Radiology, The First Affiliated Hospital of Chongqing Medical University, Chongqing, China; ^3^ Department of Radiology, The Second Hospital of Shanxi Medical University, Taiyuan, China; ^4^ Department of Radiology, Sichuan Provincial People’s Hospital, Chengdu, China

**Keywords:** machine learning, pulmonary nodules, computed tomography, pulmonary node imaging-grading reporting system, cancer imaging

## Abstract

**Purpose:**

The aim of this study was to develop and validate a prediction model for classification of pulmonary nodules based on preoperative CT imaging.

**Materials and methods:**

A data set of Centers 1 (training set: 2633; internal testing set: 1129); Center 2 and Center 3 (external testing set: 218) of patients with pulmonary nodule cases was retrospectively collected. Handcrafted features were extracted from noncontrast chest CT scans by three senior radiologists. A total of 22 clinically handcrafted parameters (age, gender, L-RADS, and PNI-GARS et al.) were used to construct machine learning models (random forest, gradient boosting, and explainable boosting) for the classification of preoperative pulmonary nodules, and the parameters of the model were adjusted to achieve optimal performance. To evaluate the prediction capacity of each model. Both 5-fold cross-validation and 10-fold cross-validation were used to test the robustness of the models.

**Results:**

The explainable boosting model had the best performance on our constructed data. The model achieves an accuracy of 89.9%, a precision of 97.48%, a specificity of 89.5%, a sensitivity of 91.1%, and an AUC of 90.3%. In human-machine comparison, the AUC of machine learning models (90.4%, 95% CI: 85.5%–94.8%) was significantly improved compared to radiologists (60%, 95% CI: 50%–71.4%).

**Conclusions:**

The explainable boosting model exhibited superior performance on our dataset, achieving high accuracy and precision in the diagnosis of pulmonary nodules compared to experienced radiologists.

## Highlights

- CT imaging features can be used to predict the benign or malignant nature of pulmonary nodules.
- Preoperative machine learning model predicts malignancy of pulmonary nodules.
- PNI-GARS enhances lung nodule diagnosis by standardizing CT grading and integrating with machine learning for improved malignancy prediction.

## Introduction

Lung cancer, the leading cause of cancer-related deaths worldwide, is responsible for a significant proportion of total cancer cases, with lung nodules often being the initial imaging manifestation of early-stage lung cancer (1–3). According to statistics released by the World Health Organization (WHO), there were approximately 2.21 million confirmed cases of lung cancer and 1.8 million deaths in 2020 (4). Lung cancer is one of the most dangerous malignancies, characterized by a poor prognosis and a low overall survival rate due to untimely detection and the limitations of conventional treatment (5, 6). The detection rate of pulmonary nodules has increased dramatically with the widespread use of multi-detector spiral CT scans. However, the majority of these nodules are benign; according to the 2011 National Lung Screening Trial (NLST), a staggering 96.4% of CT-detected lung nodules were not cancerous. Nevertheless, the presence of a lung nodule can cause significant anxiety for patients, leading to a need for accurate assessment to differentiate between malignant and benign lesions (7). The transformation of a lung nodule into lung cancer is a complex process influenced by various factors. Lung cancer development can be influenced by the size, morphology, and growth rate of the nodule. For instance, lung nodules with a diameter greater than 15 mm, those located in the upper lobe, and those exhibiting features such as spiculation, chest membrane retraction, and bronchial truncation are considered high-risk and more likely to be malignant. In the diagnosis of lung nodules, lung-RADS (Lung Imaging Reporting and Data System), a screening classification system for lung nodules, was proposed by the National College of Radiology (ACR) (8). Although Lung-RADS provide important guidance in the classification and management of pulmonary nodules, its limitations cannot be ignored, especially the lack of a comprehensive assessment of nodule imaging features such as edges, morphology, burrs, etc. The development of a lung cancer risk prediction model, therefore, presents a strategic approach to mitigate the subjectivity and unreliability inherent in radiologist diagnoses, particularly for those with limited experience (9–12). With the rapid development of artificial intelligence technology, machine learning has shown great potential in the diagnosis and treatment of lung cancer (13, 14). As the leading cause of cancer-related death worldwide, early diagnosis of lung cancer is crucial to improving treatment success and patient survival (15, 16). The machine learning model is able to identify signs of lung cancer by analyzing CT image data (17, 18). These models can automatically detect lung nodules and provide a quantitative assessment of nodule properties and can predict the histological type of lung cancer by analyzing the imaging characteristics of lung nodules, such as shape, margin, transparency, and uniformity (19–22).

The aim of this study is to incorporate a broader range of clinical and radiological features into the model, addressing the limitations of existing diagnostic systems such as the Lung-RADS classification criteria. Additionally, the developed machine learning model is comparatively analyzed with the existing Lung-RADS and PNI-GARS diagnostic systems. This comprehensive evaluation provides us with a more granular understanding of nodule characteristics and their association with malignancy. By utilizing SHAP values to explain the influence of each feature variable on the model’s output, it aids in understanding the decision-making process of the model and enhances its interpretability. Overall, this study, through the integration of multicenter data, a large sample size, advanced machine learning techniques, and comprehensive statistical analysis, has developed an efficient, accurate, and interpretable prediction model for lung nodule characterization, serving as a powerful auxiliary tool for clinical diagnosis.

## Materials and methods

### Dataset

**Figure 1:**
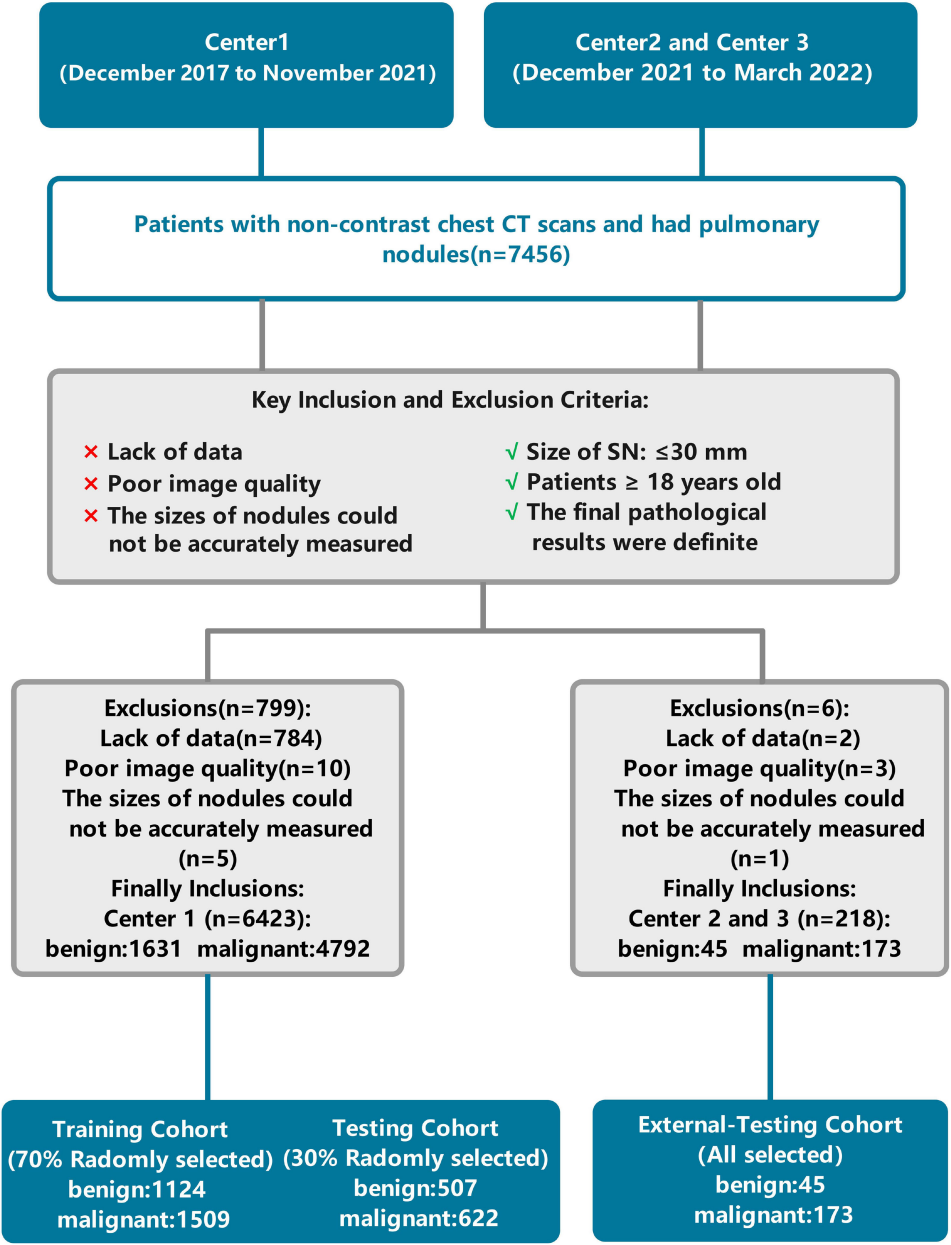
Flowchart of patient selection and data processing. Center 1, The First Affiliated Hospital of Chongqing Medical University; Center 2, Second Hospital of Shanxi Medical University; Center 3, Sichuan Provincial People’s Hospital.

The institutional review boards of the three participating institutions approved the retrospective multicohort study and waived the requirement for written informed consent. The patient data used in this study were obtained from three centers. The training set data consisted of patients from Center 1, collected between December 2017 and November 2021. Data for the external validation set were obtained from 218 patient CT examinations between December 2021 and March 2022 at Center 2 and Center 3. The inclusion and exclusion criteria were the same across all three centers. All patient data were obtained in daily practice. The inclusion criteria for the study considered the following: (1) patients aged 18 years or older; (2) size of the nodule(s) ≤ 30mm; (3) final pathological results that were definitive. The exclusion criteria for the study were as follows: (1) missing data; (2) poor image quality; (3) the size of the nodules that could not be measured accurately. The patient data selection flowchart is shown in **Figure 1**. Based on patient inclusion and exclusion criteria, 4,792 malignant and 1,631 benign pulmonary nodules from 5,404 patients at Center 1 were selected as the training set data. One hundred seventy-three malignant and 45 benign pulmonary nodules from 218 patients at Centers 2 and 3 were selected as the validation set data. To create a relatively balanced dataset for modeling, we randomly selected malignant pulmonary nodules and all benign pulmonary nodules from Center 1 at a ratio of 1.3:1 (2,131 malignant and 1,631 benign pulmonary nodules). For external validation, all pulmonary nodules from Centers 2 and 3 were included.

### Clinical features and non-contrast chest CT scans characteristics

Non-contrast chest CT scans were acquired by SOMATOM Definition Flash (Siemens Healthineers, Erlangen, Germany), SOMATOM Force (Siemens Healthineers, Erlangen, Germany), and Discovery CT750 HD (GE Healthcare, Milwaukee, WI, USA) CT scanners (**Table 1**). All patients were asked to hold their hands over their heads, lie on their backs, breathe deeply, and hold their breath. The scan range was from the tip of the lung to the level of the costophrenic angle. All images were independently and blindly read by two experienced radiologists with 8 and 10 years of experience, respectively. To assess the reliability of the readings, we calculated the agreement rate among the radiologists, which is detailed in **Supplementary Table S1**. Pathological results for each patient were collected from surgical pathology biopsies. CT features were given by each radiologist. When discrepancies occurred, the final assessment was determined by a third radiologist with 12 years of experience, who integrated the differing opinions to provide a conclusive evaluation. After a detailed evaluation, nine clinical characteristics and thirteen radiological characteristics on the CT images were used for model development. The specific features were as follows: (1) Age; (2) Sex; (3) N-nodules (Number of nodules); (4) Nature of the nodule (SN/PSN/GGN); (5) Total diameter (in mm); (6) L-RADS (1/2/3/4A/4B/4X); (7) PNI-GARS (0/I/II/IIIa/IIIb/IIIc/IV); (8) Spiculation (yes/no); (9) Lobulation (yes/no); (10) Vascular sign (yes/no); (11) Pleural indentation (yes/no); (12) Vacuole sign (yes/no); (13) Cavitations (yes/no); (14) M-features (number of malignant features); (15) Margin smooth (yes/no); (16) Pulmonary cord (yes/no); (17) Margin blurring (yes/no); (18) Calcification (yes/no); (19) Fat (yes/no); (20) Satellite feature (yes/no); (21) Nodular patchy shadow (yes/no); (22) N-features (number of benign features).

**Table 1:** The protocol parameters and reconstruction parameters for the intra-CT protocol trial.

Pitch	Tube voltage (kVp)	Tube current (mA.s)	Slice thickness (mm)	Reconstruction slice thickness(mm)	Rotation speed (s/r)	matrix
0.984	100	30/50	5	1.0	0.5/0.6	512*512
1	120	30/50	5	1.0	0.5/0.6	512*512

### Factor correlation coefficient calculation

**Figure 2:**
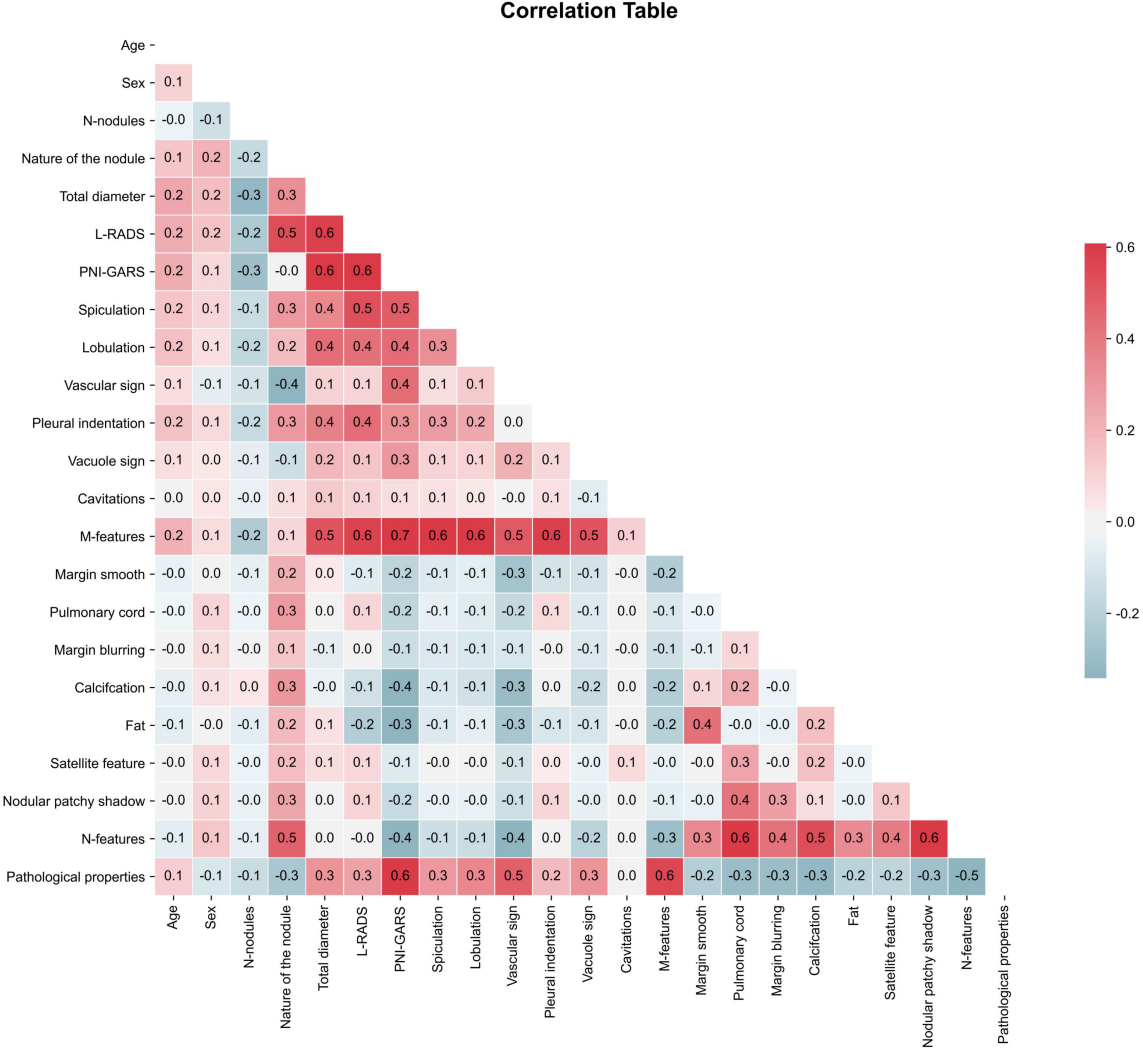
Pearson’s correlation coefficient matrix.

In the actual study, we needed to remove variables with correlation coefficients > 0.8 to prevent the occurrence of data leakage, where certain variables could directly affect the prediction results. Correlation coefficients > 0.8 indicate the presence of multicollinearity (23) in the data. In this paper, the correlation coefficient test was performed using Pearson’s coefficient (24). As shown by the statistics (**Figure 2**), all the clinical and radiological features we used had correlation coefficients not greater than 0.8, which shows that these data were suitable for machine learning model development.

### Machine learning model development

To build up a machine learning classification model for pathology prediction, we divided the pulmonary nodule data from Center 1 into a training set and an internal test set in a ratio of 7:3, and the data from centers 2 and 3 were all used in the external test set. Machine learning models used in this study included GradientBoosting, RandomForest, and ExplainableBoosting. Gradient boosting is an ensemble learning technique that iteratively trains decision trees to minimize a loss function. The advantage of the gradient boosting algorithm lies in its high-precision prediction capabilities and adaptability to various types of data. It also demonstrates superiority in situations with imbalanced data by exhibiting good robustness and generalization ability. Furthermore, the gradient boosting algorithm can assess feature importance and effectively handle large-scale datasets. Random Forest is an ensemble learning method that improves prediction accuracy and stability by constructing multiple decision trees and combining their results. Each tree in the Random Forest is trained on a randomly selected subset of samples, which reduces the variance of the model and enhances its generalization ability. The Explainable Boosting Machine (EBM) is a tree-based, iteratively gradient-boosted generalized additive model with automatic interaction detection capabilities. The design goal of EBM is to maintain comparable accuracy with state-of-the-art machine learning methods (such as Random Forest and Boosted Trees) while preserving a high degree of interpretability.

### Feature preprocessing

Feature preprocessing is a crucial step in machine learning, as it directly impacts the performance and predictive capability of the model, making the model’s predictions easier to understand and interpret. In order to enable all features to be applied for machine learning model building, we used the LabelEncoder from the scikit-learn library to perform numerical encoding on non-numerical attributes. The non-numerical characteristics were as follows: (1) Nature of the nodule; (2) L-RADS; (3) PNI-GARS. The remaining features were represented by the number 1 to indicate the presence of the imaging feature, and by the number 0 to indicate the absence of the imaging feature.

### Experimental settings

**Figure 3:**
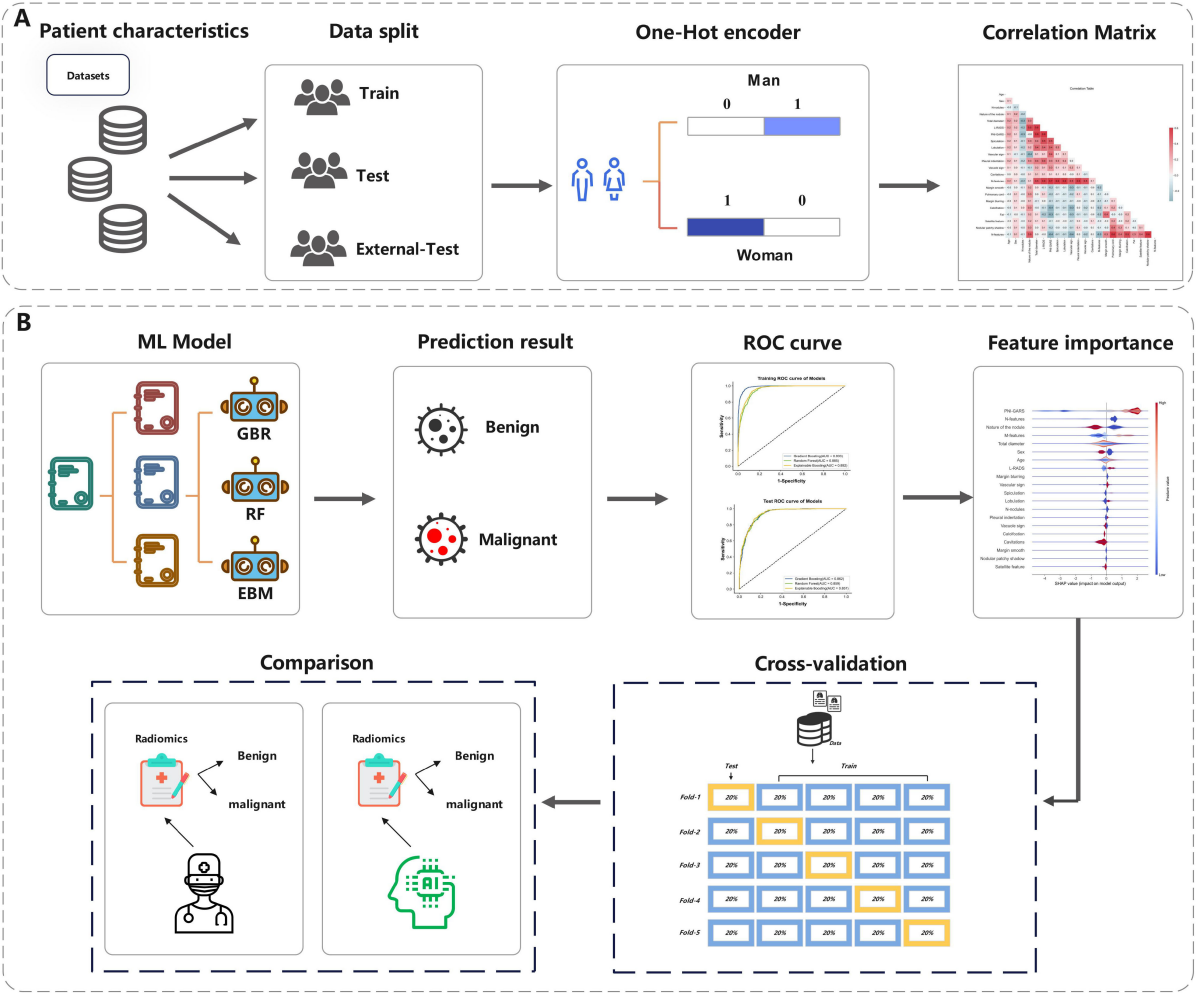
The overall pipeline of this study. **(A)** The process of data processing. **(B)** The process of Model prediction and testing.

In our study, experiments were executed using Python 3.8.3, with the experimental framework outlined in **Figure 3**. We undertook a grid search approach to hyperparameter optimization for three machine learning algorithms to achieve the best model fit for pulmonary nodule diagnosis. The GradientBoosting model yielded optimal results with a learning rate of 0.1, 140 estimators (n_estimators), a maximum tree depth of 4 (max_depth), and a minimum sample requirement for node splits of 4 (min_samples_split). For the RandomForest model, the grid search identified the most effective parameters as a maximum tree depth of 5 (max_depth), a minimum sample count at leaf nodes of 4 (min_samples_leaf), a minimum sample split of 4 (min_samples_split), 30 trees in the forest (n_estimators), and utilizing 4 jobs for parallel processing (n_jobs). Interestingly, the default parameters were found to be the most effective for the ExplainableBoosting model, indicating that the model’s designers had already established a robust starting point for a wide range of applications. Through this meticulous grid search-based optimization, we enhanced the predictive accuracy and generalizability of our models.

### Statistical analysis

The area under the receiver operating characteristic curve (AUC), accuracy, precision, sensitivity, specificity, positive predictive value (PPV) and Negative predictive value (NPV) were used to assess the diagnostic performance of the model in each cohort. Categorical variables in the patient data were represented using numerical (%) values, while continuous variables were described using means and standard deviations (SD).

## Results

### Baseline characteristics

In this study, a total of 6423 patients(Median lung nodule diameter,10.9 [IQR,7.78-29.93] mm; mean age,56.09[SD,11.13]; Man,39.4%) with CT imaging data of pulmonary nodules from three centers were analyzed according to inclusion and exclusion criteria. The training set included 2660 patients(Median lung nodule diameter,10.4 [IQR,7.2-29.76] mm; mean age,55.73[SD,10.93]; Man,40.3%).The internal test set contained 1129 patients(Median lung nodule diameter,10.4 [IQR,7.2-29.76] mm; mean age,55.73[SD,10.93]; Man,40.3%).The external test set contained 218 patients (Median lung nodule diameter,15.7 [IQR,11.4-30] mm; mean age,58.12[SD,12.17]; Man,53.1%). More detailed clinical and radiological features in different cohort are shown in **Table 2**.

**Table 2:** Clinical and non-contrast chest CT scans characteristics of patients in three cohorts.

Characteristics	Class	Total cohort (n=6423)	Training cohort (n=2633)	Internal validation cohort (n=1129)	External validation cohort (n=218)
Age, mean (SD), years	**-**	56.09 ± 11.13	55.73 ± 10.93	55.49 ± 11.08	58.12 ± 12.17
Gender, *n* (%)	Man	2532(39.4%)	1064(40.3%)	503(44.4%)	117(53.1%)
	Woman	3891(60.5%)	1569(59.5%)	626(55.3%)	101(45.9%)
Pathological properties, *n* (%)	Benign	1631(25.4%)	1124(42.6%)	507(44.9%)	45(20.4%)
	Malignant	4792(74.5%)	1509(57.2%)	622(55.0%)	173(78.6%)
*N-*nodules*.*n (%)* (1/2/3/4/5)	1	4554(70.8%)	1807(68.5%)	759(67.1%)	218(100%)
	2	1426(22.1%)	622(23.6%)	272(24.0%)	0(0%)
	3	330(5.1%)	148(5.6%)	71(6.2%)	0(0%)
	4	88(1.3%)	49(1.8%)	21(1.8%)	0(0%)
	5	25(0.3%)	7(0.2%)	6(0.5%)	0(0%)
Nature of the nodule. *n (%)* (GGN/PSN/SN)^※^	GGN	2658(41.3%)	993(37.6%)	461(40.7%)	40(18.1%)
	PSN	1516(23.5%)	513(19.4%)	214(18.9%)	48(21.8%)
	SN	2249(35.0%)	1127(42.7%)	454(40.1%)	130(59.0%)
Total diameter, median (IQR),mm	**-**	10.9(7.78-29.93)	10.4(7.2-29.76)	10.4(7.13-29.93)	15.7(11.4-30)
L-RADS. *n (%)* (1/2/3/4A/4B/4X)	1	214(3.3%)	149(5.6%)	65(5.7%)	2(0.9%)
	2	2933(45.6%)	1170(44.3%)	525(46.3%)	43(19.4%)
	3	569(8.8%)	256(9.7%)	104(9.1%)	2(0.9%)
	4A	375(5.8%)	207(7.8%)	90(7.9%)	12(5.4%)
	4B	122(1.8%)	56(2.1%)	34(3.0%)	15(6.7%)
	4X	2210(34.3%)	795(30.1%)	311(27.4%)	144(65.1%)
PNI-GARS. *n (%)* (0/II/IIIa/IIIb/IIIc/IV)	0	214(3.3%)	149(5.6%)	65(5.7%)	2(0.9%)
	I	388(6.0%)	230(8.7%)	119(9.8%)	0(0%)
	II	354(5.5%)	258(9.7%)	96(8.4%)	4(1.8%)
	IIIa	1248(19.4%)	547(20.7%)	216(19.0%)	25(11.3%)
	IIIb	830(12.9%)	309(11.7%)	135(11.9%)	34(15.1%)
	IIIc	1657(25.7%)	559(21.2%)	258(22.7%)	36(16.2%)
	IV	1732(26.9%)	581(22.0%)	247(21.8%)	117(52.9%)
Spiculation, *n* (%) (No/Yes)	**-**	4938/1485 (76.8%/23.1%)	2116/517 (80.3%/19.6%)	899/230 (97.6%/20.3%)	95/123 (43.5%/56.4%)
Lobulation, *n* (%) (No/Yes)	**-**	4008/2415 (62.3%/37.5%)	1793/840 (68.0%/31.8%)	784/345 (69.4%/30.5%)	77/141 (35.3%/64.6%)
Vascular sign, *n* (%) (No/Yes)	**-**	1196/5227 (18.6%/81.3%)	701/1932 (25.6%/73.3%)	307/822 (27.1%/72.8%)	97/121 (44.4%/55.5%)
Pleural indentation, *n* (%) (No/Yes)	**-**	4303/2120 (65.9%/32.9%)	1833/800 (69.5%/30.3%)	803/326 (71.1%/28.8%)	104/114 (47.7%/52.2%)
Vacuole sign, *n* (%) (No/Yes)	**-**	4333/2090 (67.4%/32.5%)	1907/726 (72.3%/27.5%)	837/292 (74.1%/25.8%)	129/89 (59.1%/40.8%)
Cavitations, *n* (%) (No/Yes)	**-**	6322/101 (98.3%/1.5%)	2597/36 (98.5%/1.3%)	1109/20 (98.2%/1.7%)	182/35 (83.4%/16.5%)
M-Features^Ꭽ^. *n (%)* (0/1/2/3/4/5/6)	0	562(8.7%)	385(14.6%)	165(14.6%)	5(2.2%)
	1	1697(26.4%)	766(29.0%)	357(31.6%)	41(18.8%)
	2	1922(29.9%)	735(27.8%)	292(25.8%)	39(17.8%)
	3	1296(20.1%)	441(16.7%)	199(17.6%)	48(22.0%)
	4	722(11.2%)	238(9.0%)	83(7.3%)	46(21.1%)
	5	223(3.4%)	68(2.5%)	33(2.9%)	31(14.2%)
	6	1(0.01%)	0(0%)	0(0%)	8(3.6%)
Margin smooth, *n* (%) (No/Yes)	**-**	6258/165 (97.6%/2.5%)	2526/107 (95.8%/4.0%)	1079/50 (95.5%/4.4%)	218/0 (100%/0%)
Pulmonary cord, *n* (%) (No/Yes)	**-**	6131/292 (95.4%/4.5%)	2420/213 (91.8%/8.1%)	1059/70 (93.7%/6.2%)	214/4 (98.1%/1.8%)
Margin blurring, *n* (%) (No/Yes)	**-**	6175/248 (96.1%/3.8%)	2469/164 (93.7%/6.2%)	1047/82 (92.7%/7.2%)	218/0 (100%)
Calcification, *n* (%) (No/Yes)	**-**	6125/298 (95.3%/4.8%)	2426/207 (92.0%/8.0%)	1042/86 (92.3%/7.6%)	213/5 (97.7%/2.2%)
Fat, *n* (%) (No/Yes)	**-**	6318/105 (98.3%/1.6%)	2563/70 (97.2%/3.8%)	1094/35 (96.8%/3.1%)	218/0 (100%/0%)
Satellite feature, *n* (%) (No/Yes)	**-**	6327/96 (98.5%/1.5%)	2571/62 (97.5%/2.3%)	1097/32 (97.1%/2.8%)	212/6 (97.2%/6%)
Nodular patchy shadow, *n* (%) (No/Yes)	**-**	6128/4.5 (95.4%/4.5%)	2438/195 (92.5%/7.4%)	1049/80 (92.9%/7.0%)	210/8 (96.3%/3.6%)
N-Features^ჭ^, *n* (%) (0/1/2/3/4)	0	5457(84.9%)	1995(75.7%)	842(74.5%)	197(90.3%)
	1	534(8.3%)	330(12.5%)	168(14.8%)	19(8.7%)
	2	338(5.2%)	241(9.1%)	72(8.1%)	2(0.9%)
	3	87(1.3%)	62(2.3%)	25(2.2%)	0(0%)
	4	7(0.1%)	5(0.1%)	2(0.1%)	0(0%)

*Total number of pulmonary nodules, ※GGN, Ground glass nodule; PSN, Part-solid nodule; SN, Solid nodule; ᎭM-Features, Number of malignant features; ჭN-Features, Number of benign features.

### Machine learning model performance

**Figure 4:**
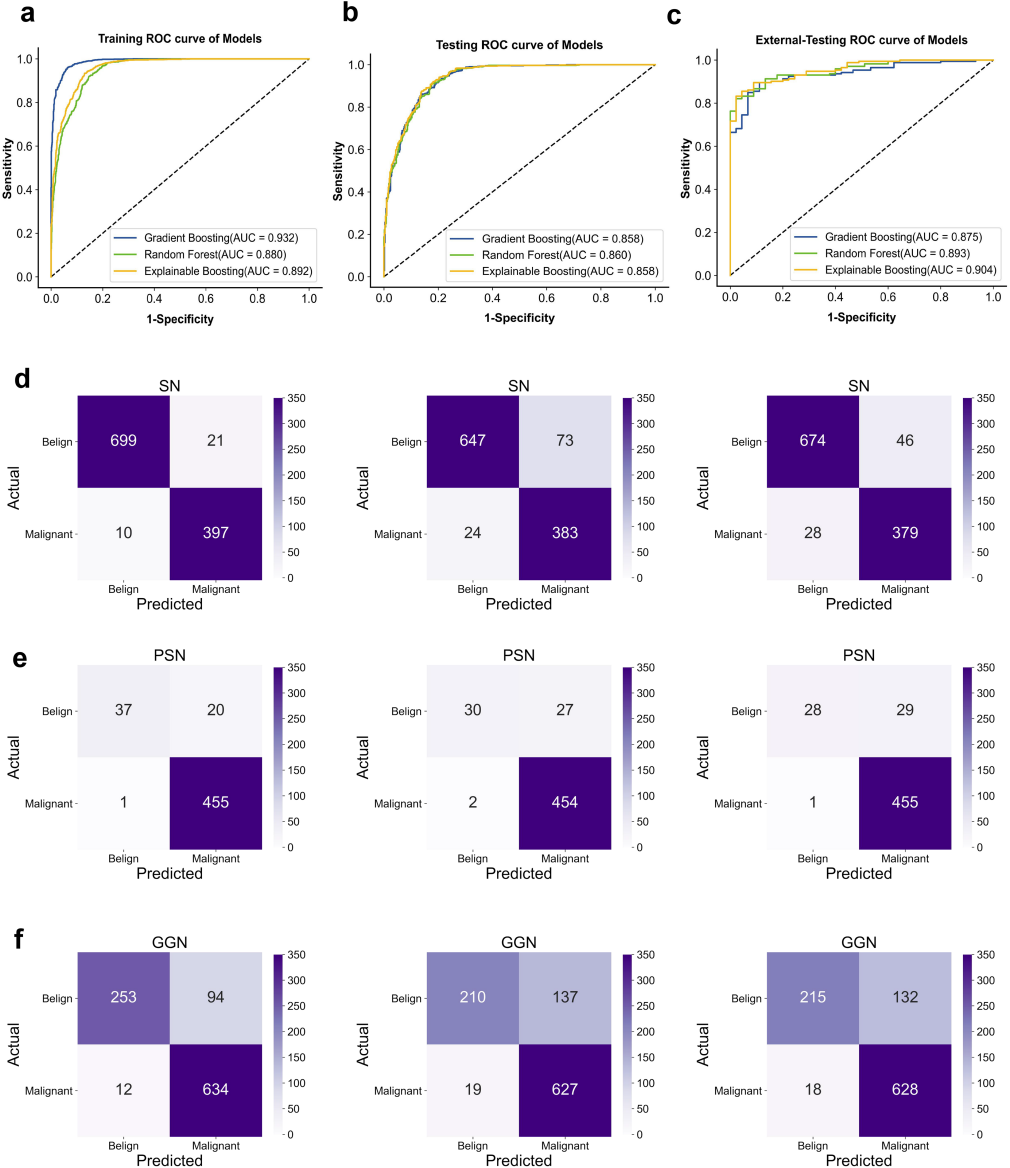
ROC curve for classification in the dataset. **(A)** training-sets. **(B)** internal-testing-sets. **(C)** external-testing-sets; AUC, area under curve. Confusion matrix. **(D)** Classification performance of different models on SN. **(E)** PSN. **(F)** GGN-type nodules (From left to right; Gradient Boosting; Random forest; Explainable Boosting.

During the training process, different machine learning models showed different performance on clinical-radiological features. In the external-testing set, Explainable Boosting showed the best fitting results with an AUC of 0.904 (95% CI: 0.855–0.948), accuracy of 0.899, sensitivity of 0.911, specificity of 0.895, PPV of 0.974, and NPV of 0.694. While in the internal-testing set, these values were 0.858 (95% CI: 0.839–0.877), 0.867, 0.767, 0.948, 0.833, and 0.923, respectively. Furthermore, Explainable Boosting compared favorably to the Random Forest model and the Gradient Boosting model, with sensitivity improvements of 0.089 and 0.023, respectively. Specificity was 0.034 higher compared to the Gradient Boosting model. The prediction performance of the model across three cohorts is shown in **Figures 4A–C** and **Table 3** . By calculating the confusion matrix, the values of True Positive (TP), False Positive (FP), True Negative (TN), and False Negative (FN) (25) can be obtained. The five metrics used to evaluate our model are as follows:

1. Accuracy=(TP + TN)/(TP + TN + FP + FN).
2. Sensitivity=TP/(TP + FN).
3. Specificity=TN/(TN + FP).
4. PPV=TP/(TP + FP).
5. NPV=TN/(FN + TN).

**Table 3:** The performance comparison of different models.

Model	Cohort	AUC(95%CI)	Accuracy	Sensitivity	Specificity	PPV	NPV
Gradient Boosting	Training	0.932 [0.922-0.942]	0.903 [0.847-0.935]	0.879 [0.835-0.899]	0.984 [0.968-0.991]	0.916 [0.882-0.933]	0.977 [0.941-0.980]
	Internal testing	0.858 [0.837-0.878]	0.865 [0.844-0.892]	0.790 [0.751-0.833]	0.926 [0.888-0.935]	0.844 [0.801-0.891]	0.897 [0.823-0.915]
	External testing	0.875 [0.821-0.925]	0.866 [0.831-0.901]	0.888 [0.784-0.902]	0.861 [0.844-0.886]	0.967 [0.903-0.985]	0.625 [0.554-0.701]
RandomForest	Training	0.880 [0.868-0.893]	0.894 [0.867-0.911]	0.790 [0.788-0.835]	0.971 [0.951-0.991]	0.861 [0.812-0.889]	0.953 [0.901-0.986]
	Internal testing	0.860 [0.838-0.879]	0.870 [0.842-0.903]	0.753 [0.701-0.855]	0.966 [0.953-0.979]	0.827 [0.787-0.865]	0.947 [0.899-0.967]
	External testing	0.893 [0.806-0.925]	0.899 [0.855-0.932]	0.822 [0.786-0.856]	0.919 [0.875-0.935]	0.952 [0.899-0.978]	0.725 [0.621-0.832]
ExplainableBoosting	Training	0.892 [0.880-0.904]	0.903 [0.875-0.935]	0.815 [0.789-0.866]	0.968 [0.922-0.989]	0.875 [0.833-0.896]	0.951 [0.901-0.987]
	Internal testing	0.858 [0.839-0.877]	0.867 [0.841-0.891]	0.767 [0.715-0.864]	0.948 [0.901-0.963]	0.833 [0.791-0.881]	0.923 [0.878-0.943]
	External testing	0.904 [0.855-0.948]	0.899 [0.888-0.925]	0.911 [0.894-0.935]	0.895 [0.881-0.922]	0.974 [0.945-0.989]	0.694 [0.644-0.721]

PPV, positive predictive value; NPV, negative predictive value; CI, confidence interval.

### Classification effectiveness assessment

In the second phase of experiments, we demonstrated the performance of machine learning in classifying three types of pulmonary nodule properties: SN (solid nodule), PSN (partially solid nodule), and GGN (ground glass nodule). The average probability of misclassifying SN, PSN, and GGN-type pulmonary nodules as malignant or benign by the three models was 2.9%, 4.0%, and 6.8%, respectively. The average probability of misclassifying benign as malignant was 10.6%, 5.2%, and 16.0%, respectively. Of the three types of nodules, the model had the best performance in diagnosing the SN type of nodule, and the GGN type had the worst performance. In clinical practice, the probability of malignancy is, in descending order, PSN > GGN > SN. However, the probability of misclassifying malignant as benign is on average 6.0% lower in our model than the probability of misclassifying benign as malignant. Specific classification details are shown in **Figures 4D–F**. Therefore, we calculated diagnostic precision and recall scores separately for male and female patients in the dataset to compare the diagnostic performance of the model across genders. The details of the relevant scores are shown in **Supplementary Figure S2A, B**. The diagnostic performance of male patients is slightly better than that of female patients on the external validation set. However, there were fewer benign cases in the external validation set, which resulted in a lower recall score. The diagnosis of benign nodules by radiologists is higher than our model. Thus, the use of machine learning can assist in improving the diagnostic accuracy of pulmonary nodule pathology by radiologists. In addition, we found that the diagnostic performance of the model continued to improve with age by exploring the diagnostic performance at different ages. It indicates that our model has a significant impact on the diagnosis of malignant lung nodules in the elderly population. The age-specific diagnostic information is shown in **Supplementary Figure S2C**.

### Model validation

In this study, we used 5-fold cross-validation and 10-fold cross-validation to test the stability of the machine learning model, respectively. The cross-validation process is shown in **Supplementary Figure S1A**. Cross-validation helps to mitigate potential bias and overfitting problems that can arise from using a single training-testing split. In addition, the cross-validated classification results of different models are shown in **Supplementary Figures S1B, C**.

In the 5-fold and 10-fold cross-validation used for the different cohorts of patient data, the three machine learning models performed stable AUC values in the training set and in the internal test set. but the accuracy values fluctuate more on the external validation set data, which may be due to the non-uniformity of the benign and malignant samples in the external validation set. and with uneven dichotomous samples, the AUC and precision metrics are more representative of the overall prediction.

### Model feature interpretability

**Figure 5:**
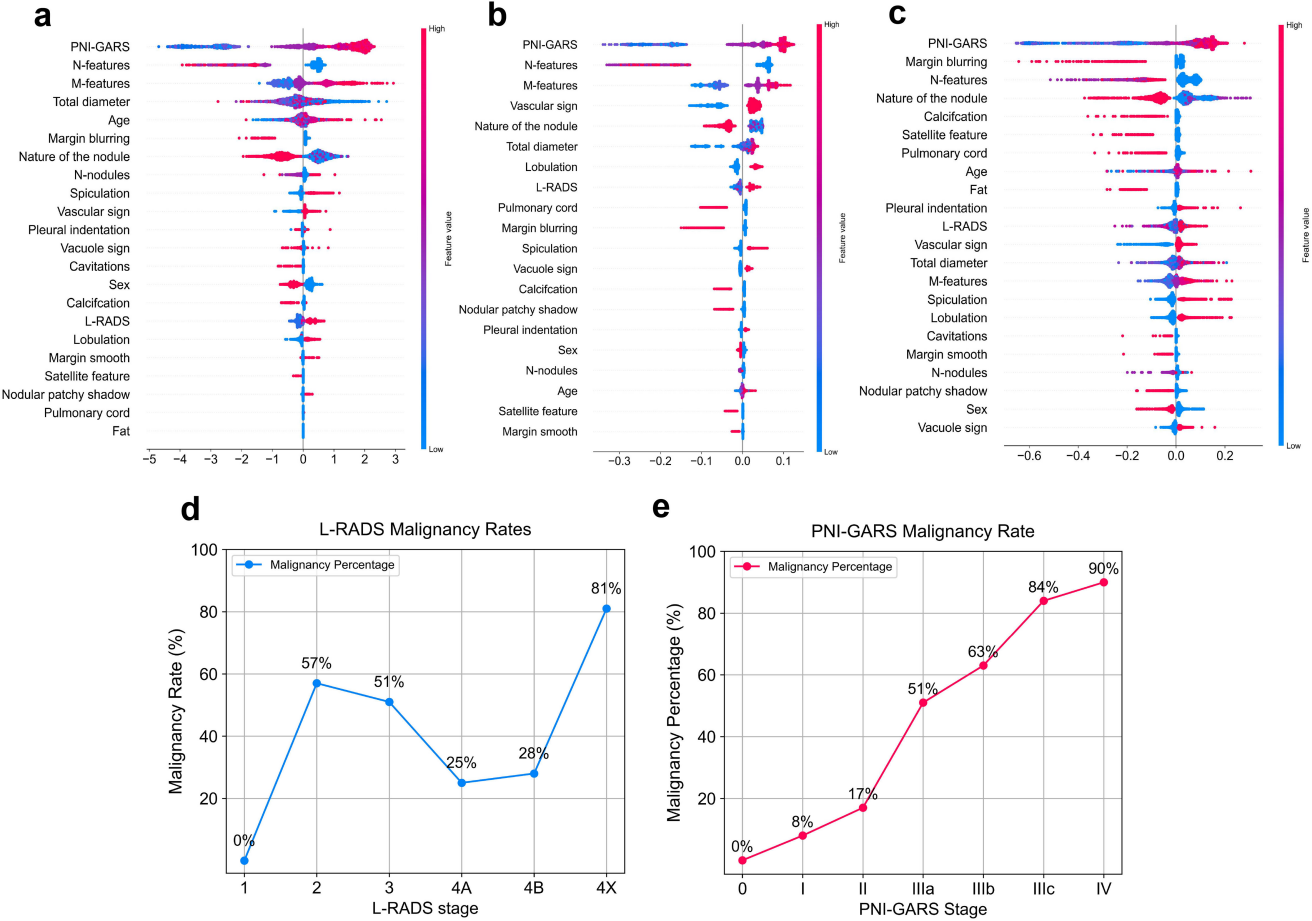
Feature contributions of different machine learning models. **(A)** Gradient Boosting. **(B)** Randomforest. **(C)** Explainable Boosting. Diagnostic accuracy of the pulmonary nodule classification system for malignant nodules at different stages. **(D)** L-RADS system. **(E)** .PNI-GARS system.

**Figure 6:**
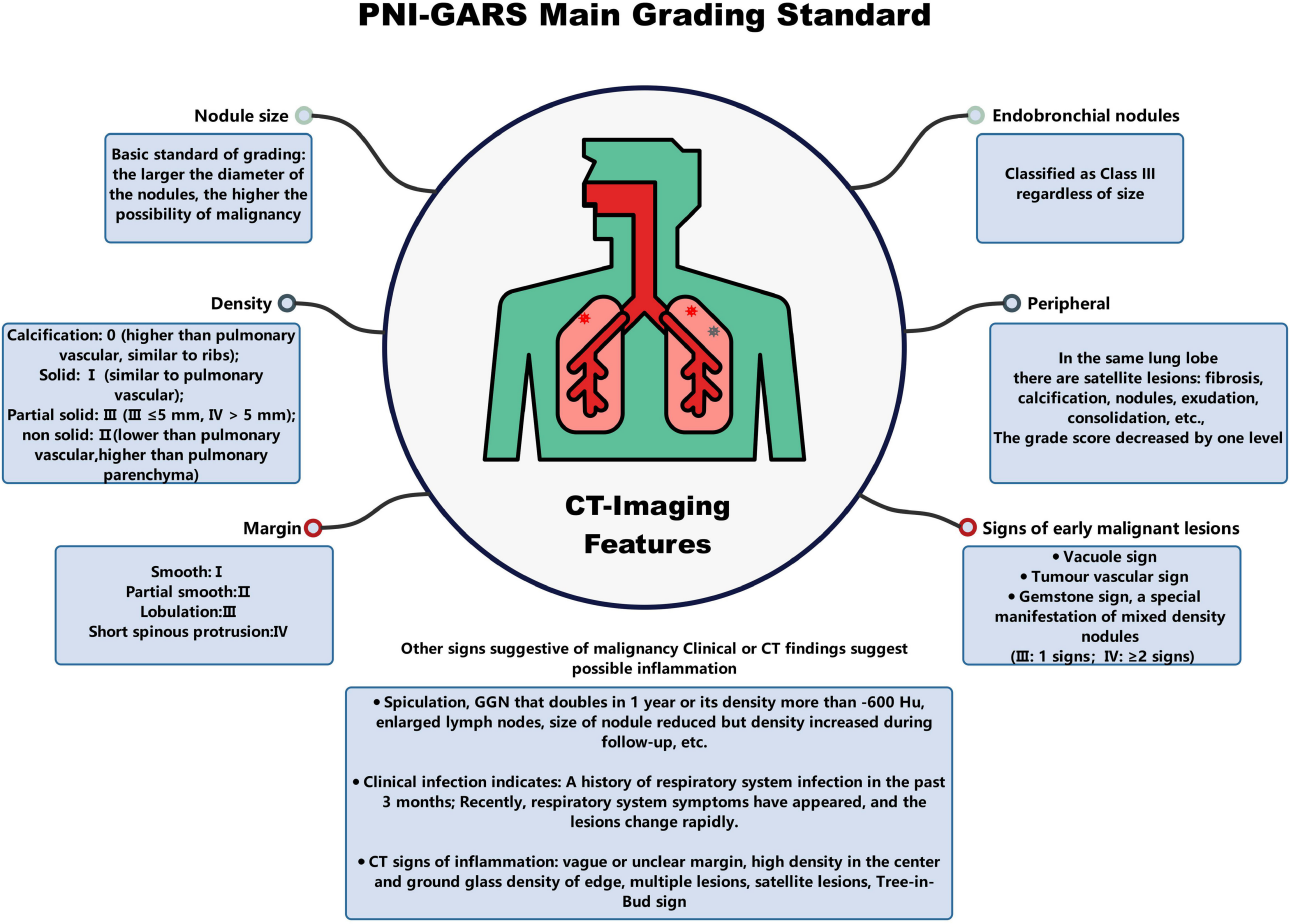
Main grading basis of PNI-GARS.

The SHAP values (26) elucidate the influence of each feature variable on the prediction model output (**Figure 5**). The importance of features is depicted with a decreasing gradient from top to bottom. In the context of this study, “positive samples” refer to malignant lung nodules, while “negative samples” denote benign nodules. A red color signifies a greater impact on the classification of malignant nodules (positive samples), whereas a blue color indicates a greater impact on the classification of benign nodules (negative samples). The x-axis represents the SHAP value, where positive values suggest a contribution towards a positive classification (malignant), and negative values imply a negative impact on this classification, potentially leaning towards a negative classification (benign). The interaction of features with negative values in the prediction is contingent upon the interplay with other variables. Wider bars on the plot indicate higher density and more recurrent values. As depicted in **Figures 5A–C**, distinct machine learning models prioritize features differently. We identified the top five features for prediction in each model (**Supplementary Table S2**). PNI-GARS was identified by three machine learning models as the primary indicator of benign and malignant pulmonary nodules. Upon comparing the diagnostic efficacy of the PNI-GARS and the L-RADS systems for malignant lung nodules using identical patient data, the L-RADS system was observed to be less effective in diagnosing malignant lung nodules across various stages. Conversely, the PNI-GARS system demonstrated incremental improvements in diagnostic accuracy for malignant lung nodules with each progressive grading level. The detailed diagnostic performance, which included data from all cohorts—the training set, the validation set, and the external validation set—is illustrated in **Figures 5D, E**. The PNI-GARS system thus offers superior diagnostic efficacy for lung nodule assessment compared to the L-RADS system. The specific PNI-GARS grading criteria are outlined in **Table 4** and depicted in **Figure 6**.

**Table 4:** PNI-GARS.

Category	Grade	Characteristics	Probability of malignancy
Definitely benign	0	No lung nodules/Pure-calcified nodules Fatty nodules, spherical atelectasis, interlobar fissure nodules, etc.	–
Benign	I	Micronodules: Any density ≤ 5 mm. Solid nodules >5 mm,&stable≥2 years on follow-up. Sub-solid nodules>5 mm,&stable≥5 years(with decrease but no increase in density or disappearance) on follow-up.	–
Probably Benign	II	Acinus nodule:5-8mm&margins partially smooth	Very low
Probably Malignant	III	Any nodule size: 8-30 mm (≤30 mm). Partial solid nodules: gemstone sign & Solid component ≤ 5 mm. Endobronchial nodules.	–
	IIIa	Small-size nodules: 8-10 mm. Nodule size ≤ 8 mm, Grade II nodules with malignant features such as gemstones, vacuoles, tumor vessels, lobulation, etc.	Medium
	IIIb	Medium-size nodules: 10-20 mm. Any nodule size ≤ 10 mm, Grade IIIa nodules with malignant features such as gemstones, vacuoles, tumor vessels, lobulation, etc.	High
	IIIc	Large-size nodules:20-30 mm. Any nodule size ≤ 10 mm, Grade IIIb nodules with malignant features such as gemstones, vacuoles, tumor vessels, lobulation, etc.	Very high
Highly suspicion of malignancy	IV	Any nodule size:8-30 mm(with spiculation sign or vacuoles, tumor vessels, lobulation, etc). Partial solid nodules (gemstone feature, solid composition> 5 mm).	Malignancy confirmed by imaging
Pathologically confirmed malignant	V	Malignancy was confirmed by pathology.	Malignancy confirmed

### Diagnostic performance of the radiologist and machine learning model

A comparison of diagnoses between radiologists and machine learning models is presented in **Table 5**. In the external validation set, the radiologist with 8 years of clinical experience, Radiologist A, achieved a performance with an AUC of 0.60 (95% CI: 0.500–0.714), accuracy of 0.707, sensitivity of 0.200, specificity of 0.993, positive predictive value (PPV) of 0.684, and negative predictive value (NPV) of 0.986. Another radiologist with 10 years of clinical experience, Radiologist B, demonstrated performance metrics: AUC of 0.720 (95% CI: 0.611–0.792), accuracy of 0.751, sensitivity of 0.552, specificity of 0.982, PPV of 0.754, and NPV of 0.991. Upon analyzing the performance metrics of the radiologists and the machine learning models, it is evident that the machine learning models demonstrate superior diagnostic performance in the classification of pulmonary nodules. Specifically, the Gradient Boosting model achieved an AUC of 0.875 (95% CI: 0.821–0.925), accuracy of 0.866, sensitivity of 0.888, specificity of 0.861, PPV of 0.967, and NPV of 0.625. The Random Forest model showed slightly higher diagnostic accuracy with an AUC of 0.893 (95% CI: 0.806–0.925), accuracy of 0.899, sensitivity of 0.822, specificity of 0.919, PPV of 0.952, and NPV of 0.725. The Explainable Boosting model led the comparison with the highest AUC of 0.904 (95% CI: 0.855–0.948), accuracy of 0.899, sensitivity of 0.911, specificity of 0.895, PPV of 0.974, and NPV of 0.694. The Explainable Boosting model shows high sensitivity and NPV. On the other hand, the specificity and PPV values from the radiologists’ diagnoses indicate a high likelihood of correctly identifying patients with benign nodules. Radiologist A achieved a specificity of 0.993 and a PPV of 0.684, while Radiologist B obtained a specificity of 0.982 and a PPV of 0.754. However, when it comes to sensitivity, the data suggest that the machine learning models are more effective in identifying actual cases of malignancy. Radiologist A had a sensitivity of 0.200, and Radiologist B had a sensitivity of 0.552, which, while improved over A, still fell short of the machine learning models’ sensibilities. This highlights the importance of machine learning models in enhancing the detection of malignant nodules. For the remaining malignant data after balancing the data set, we combine this data with benign data to form a new data set, and predict this new data set by using ExplainableBoosting model to evaluate the performance of the model. The relevant prediction results are shown in **Supplementary Tables S3**, **S4**. ExplainableBoosting still showed good classification performance in the remaining data, with an AUC value of 0.876(95% CI: 0.857-0.895).The AUC curve is shown in **Supplementary Figure S3**. For malignant nodules, the accuracy and recall rates were 0.97 and 0.92, respectively, and for benign nodules, the accuracy and recall rates were 0.94 and 0.79, respectively. This means that the model can identify malignant nodules accurately and comprehensively. However, when it comes to predicting benign nodules, while accuracy is high, recall rates are relatively low.

**Table 5:** Performance comparison between machine learning model and Radiologist in external-validation set.

Model	AUC(95%CI)	Accuracy	Sensitivity	Specificity	PPV	NPV
Gradient Boosting	0.875 [0.821-0.925]	0.866 [0.831-0.901]	0.888 [0.784-0.902]	0.861 [0.844-0.886]	0.967 [0.903-0.985]	0.625 [0.554-0.701]
Random Forest	0.893 [0.806-0.925]	0.899 [0.855-0.932]	0.822 [0.786-0.856]	0.919 [0.875-0.935]	0.952 [0.899-0.978]	0.725 [0.621-0.832]
Explainable Boosting	0.904 [0.855-0.948]	0.899 [0.888-0.925]	0.911 [0.894-0.935]	0.895 [0.881-0.922]	0.974 [0.945-0.989]	0.694 [0.644-0.721]
Radiologist A	0.600 [0.500-0.714]	0.707 [0.610-0.751]	0.200 [0.174-0.353]	0.993 [0.901-1.000]	0.684 [0.593-0.755]	0.986 [0.905-0.993]
Radiologist B	0.720 [0.611-0.792]	0.751 [0.699-0.830]	0.552 [0.435-0.699]	0.982 [0.956-1.000]	0.754 [0.674-0.815]	0.991 [0.965-1.000]

PPV, positive predictive value; NPV, negative predictive value; CI, confidence interval.

## Discussion

In our study, we found that the machine learning model we developed were highly accurate for pulmonary nodule diagnosis. The PNI-GARS system were all recognized by the three machine learning models as a first indicator of the influence of the benign versus malignant nature of lung nodules. In contrast, the Lung-RADS classification criteria, which are now widely used internationally, were less effective in classifying lung nodules as benign or malignant in our dataset. The results suggest that our use of clinical patient characteristics and the PNI-GARS grading system can help radiologists improve the accuracy of their diagnosis of pulmonary nodules.

Currently, research is focused on how CT can be used to achieve an accurate diagnosis of pulmonary nodules without interventional procedures. The Mayo Clinic model, the Veterans Affairs (VA) model, the Brock model (PanCan model), and the Herder model were widely used for pulmonary nodule malignancy diagnosis (19, 20, 27). However, the above studies suggest that these models have limited performance in the clinical prediction of malignant lung nodules (28–30). Moreover, these models were performed with data from lung cancer screening trials, where the majority of patients were clinically asymptomatic and more benign. In contrast, the model we developed was based on a wider range of patient CT imaging presentations and combined two pulmonary nodule diagnostic systems for lung nodules, which were comprehensively evaluated in patients, validated with internal data as well as cross-center validation. In the same data situation, the models proposed in previous studies and the diagnoses made by radiologists were compared, our model had a predictive accuracy with an AUC of 90.3% (CI: 85.5%-94.8%), which was not only higher than that of radiologists with 60% (CI: 50.0%-71.4%), but also higher than that of Mayo, (74.5%; 95% CI: 71.8%-81.5%); Brock, (78.3%, 95% CI: 71.5% -83.8%); VA, (70%, 95% CI 65.5%-71.4%).

As artificial intelligence continues to develop, machine learning is also commonly used in the analysis of medical data. Machine learning algorithms specialize in discovering associations between data rather than the one-dimensional statistical methods currently used (31) (e.g., logistic regression). As computing power and storage continue to increase, machine learning algorithms are able to analyze more complex data and make decisions faster (32, 33). A one-dimensional logistic model was used to predict cancer classification, a traditional approach in the study by Cui X et al (34, 35). Our research uses multiple integrated learning models to fit the data and makes the machine learning decision-making process more transparent by outputting a ranking of model features. Random Forest classification is a method that combines several randomly selected trees and makes predictions by averaging them. This method is of great interest to the research community due to its high accuracy, superiority, and improved performance (36). The gradient boosting method can capture complex relationships in clinical research better than methods based on generalized linear models (37). Magunia H et al. stratified patient risk and predicted ICU survival and prognosis by developing a machine learning model(ExplainableBoosting) based on retrospective and prospective clinical data (38). In this paper, all three machine learning models performed well in terms of classification predictive results in the medical data.

In addition, the PNI-GARS system is proposed on the premise of standardizing the writing of CT reports on lung cancer and grading different nodules, classifying nodules into grades 0 to V. As the grading level increases, the risk of malignancy of the nodules increases, and the different grades of nodules are closely related to the next step of the diagnostic and therapeutic protocols. However, we found that the PNI-GARS system is limited by combining all features into one level. So, we used machine learning to combine clinical and radiological features and the PNI-GARS system to obtain more accurate predictions.

There are several limitations of this study, the first is that our model did not take into account additional clinical indicators of the patient such as smoking history, living environment, family history of cancer, work environment, and previous history of cancer, etc., and the VA model used smoking history and history of cancer to determine the malignancy of lung nodules. However, even in the case where we did not use these indicators, the prediction performance of our model was still higher than that of the VA model. Secondly, the samples selected for our model were surgically confirmed disease cases, which may have been treated surgically with a high degree of suspicion of malignancy by the radiologist, which may be biased. Thirdly, the small number of cases in the external dataset phase of the multicenter study meant that we did not search for cases with two pulmonary nodules or more at the same time. In subsequent studies, we will include as many more clinical factors as possible as well as life factors of the patients and apply the model to cases that were not involve surgical treatment to validate the validity of the model.

To conclude, we selected the machine learning model by analyzing the best results obtained in the previous studies, combining it with our self-developed PNI-GARS system and clinical characterization data, and validating it with data from different centers, resulting in excellent predictions of the nature of pulmonary nodules. This demonstrates that by combining the PNI-GARS system with clinical imaging features and using machine learning to predict the nature of lung nodules, it can be used to clinically assist radiologists in pathological diagnosis.

## Data Availability

The raw data supporting the conclusions of this article will be made available by the authors, without undue reservation.
