# Supplementary material for: Prediction of benign and malignant pulmonary nodules using preoperative CT features: using PNI-GARS as a predictor
Source: Front Immunol. 2024 Nov 20;15:1446511. doi: 10.3389/fimmu.2024.1446511 (PMC11614820; doi:10.3389/fimmu.2024.1446511)
Supplement: Supplementary file 1 [file DataSheet1.docx]

Prediction of benign and malignant pulmonary nodules using preoperative CT features:Using PNI-GARS as a predictor

Supplementary Material


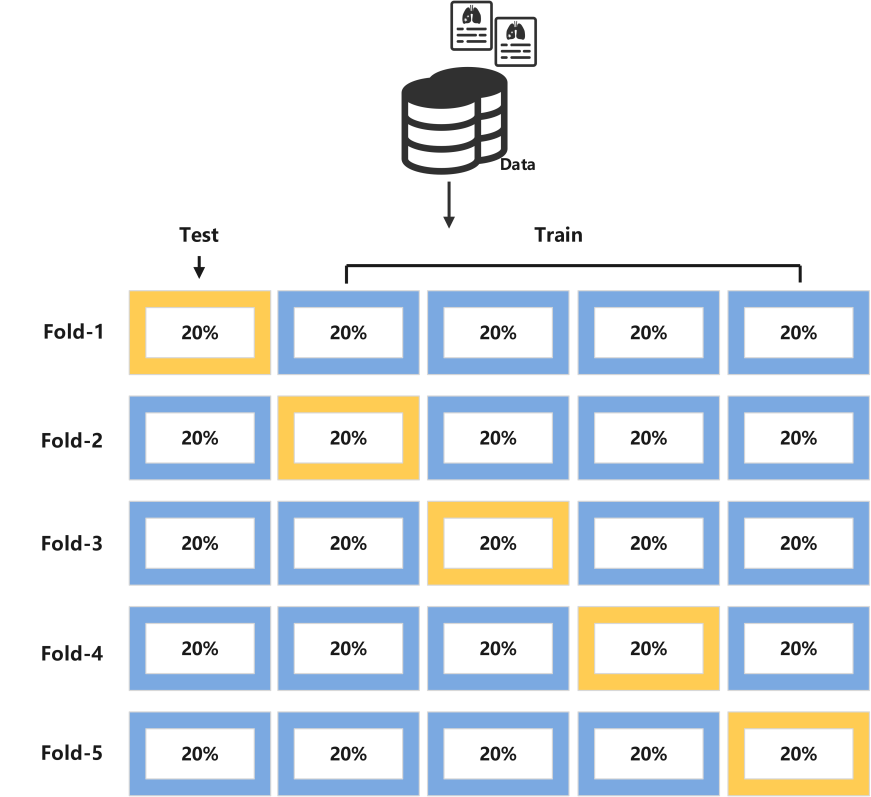


**a**


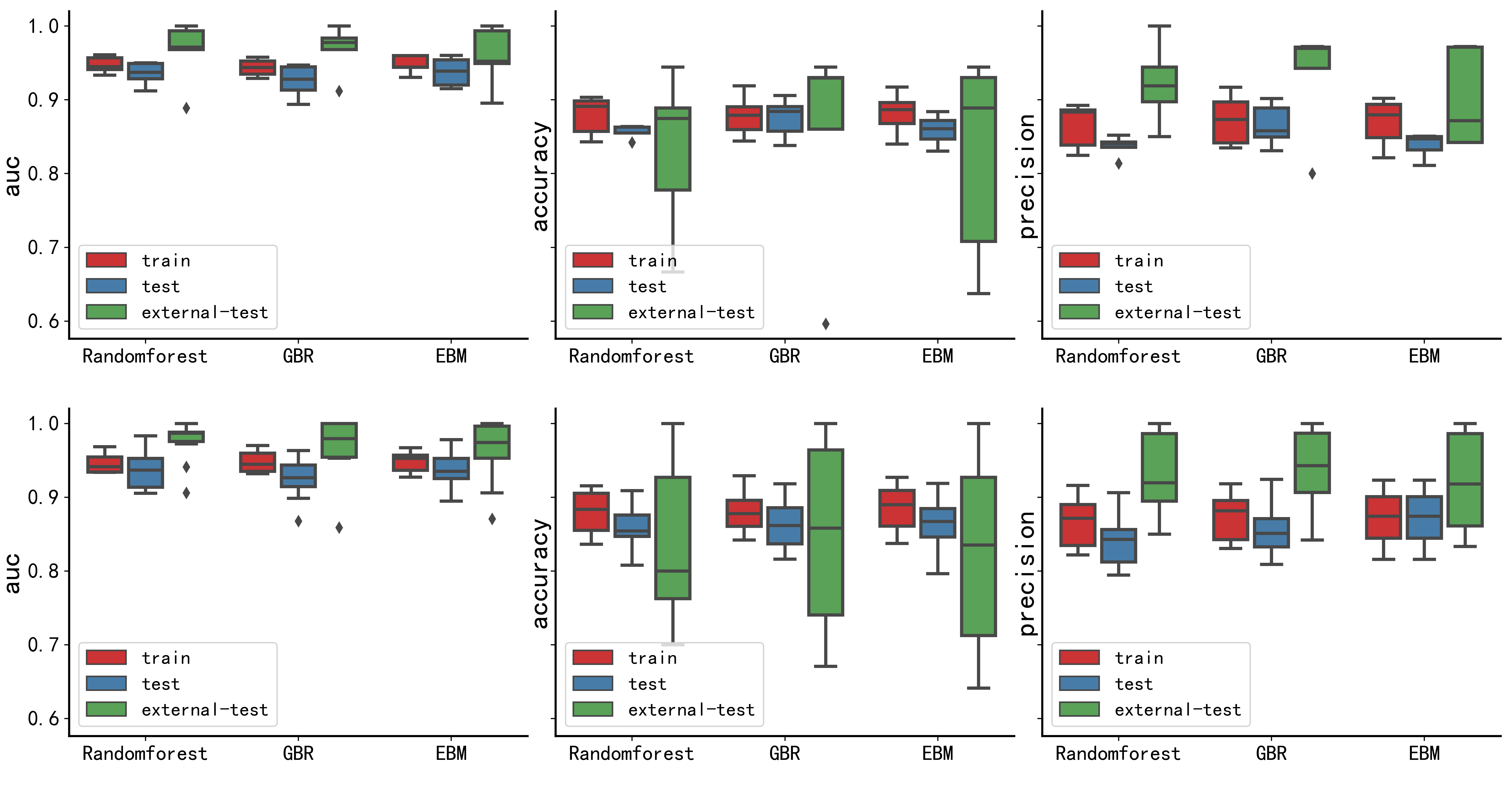


**b**

**c**

**FIGURE S1.Cross-validation process. a.**cross-validation processing. **b**.AUC, accuracy, and precision performance of the three models on 5-fold cross-validation **c.**AUC, accuracy, and precision performance of the three models on 10-fold cross-validation.

**b**

**a**


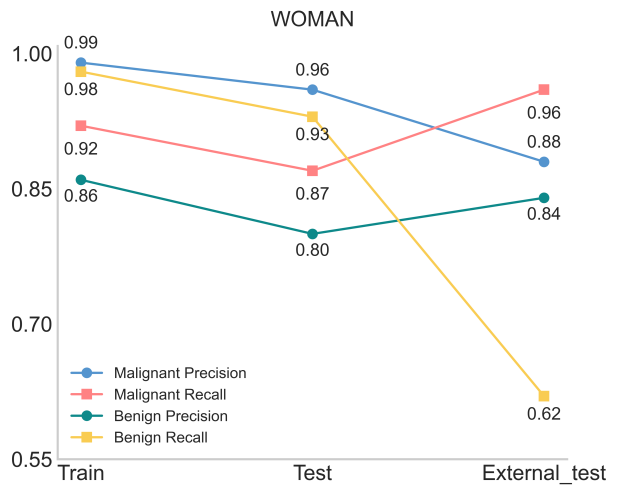

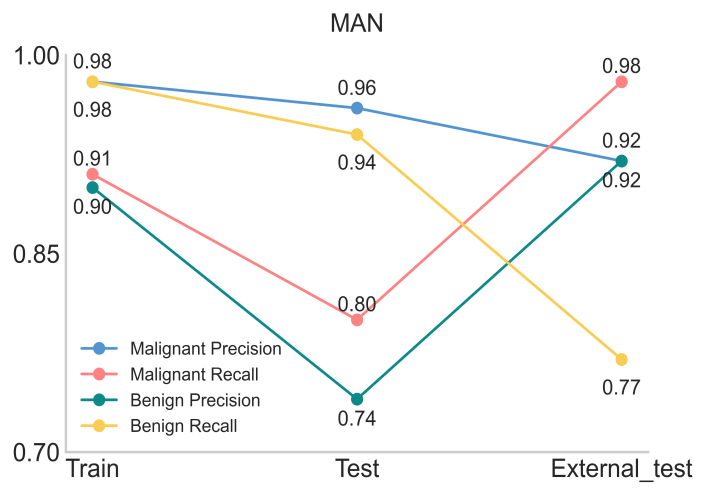


**c**

**
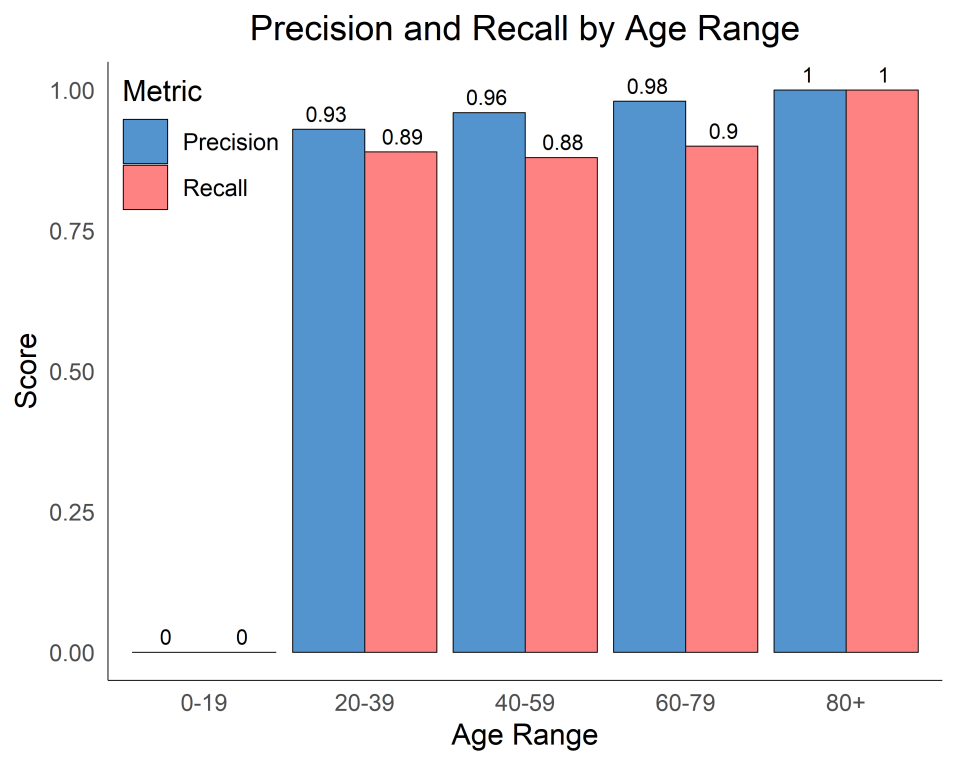
**

**FIGURE S2.Analysis of beneficiary populations.**Precision and recall scores for diagnosis of benign and malignant lung nodules in different cohorts.**a**.Male patients.**b**.Fmale patients. **c**.Diagnostic Precision and Recall Scores for Patients of Different Ages.

| **Table s1** Agreement rate among different radiologists | | | |
| --- | --- | --- | --- |
| Features^*^ | AR(%)(95%CI) | Features^▲^ | AR(%)(95%CI) |
| Spiculation^[1]^ | 0.95[0.932-0.966] | Margin smooth^[4]^ | 0.98[0.973-0.991] |
| Lobulation^[2]^ | 0.93[0.915-0.958] | Pulmonary cord^[4]^ | 0.92[0.918-0.936] |
| Vascular sign^[3]^ | 0.97[0.963-0.988] | Margin blurring^[4]^ | 0.96[0.955-0.975] |
| Pleural indentation^[3]^ | 0.91[0.899-0.935] | Fat^[4]^ | 0.93[0.918-0.945] |
| Vacuole sign^[3]^ | 0.90[0.894-0.915] | Satellite feature^[4]^ | 0.97[0.946-0.989] |
| Cavitations^[3]^ | 0.95[0.935-0.969] | Nodular patchy shadow^[4]^ | 0.95[0.941-0.978] |

| **Table s2** Top 5 features in different machine learning models | | | | | |
| --- | --- | --- | --- | --- | --- |
| Model | Top 1 | Top 2 | Top 3 | Top 4 | Top 5 |
| Gradient Boosting | **PNI-GARS** | **N-features** | **M-features** | Toal diameter | Age |
| RandomForest | **PNI-GARS** | **N-features** | **M-features** | Vascular sign | Nature of nodule |
| ExplainableBoosting | **PNI-GARS** | Margin blurring | **N-features** | Nature of nodule | Calcifcation |

Abbreviations:AR:agreement rate;CI:confidence interval.*malignant sign ▲benign sign


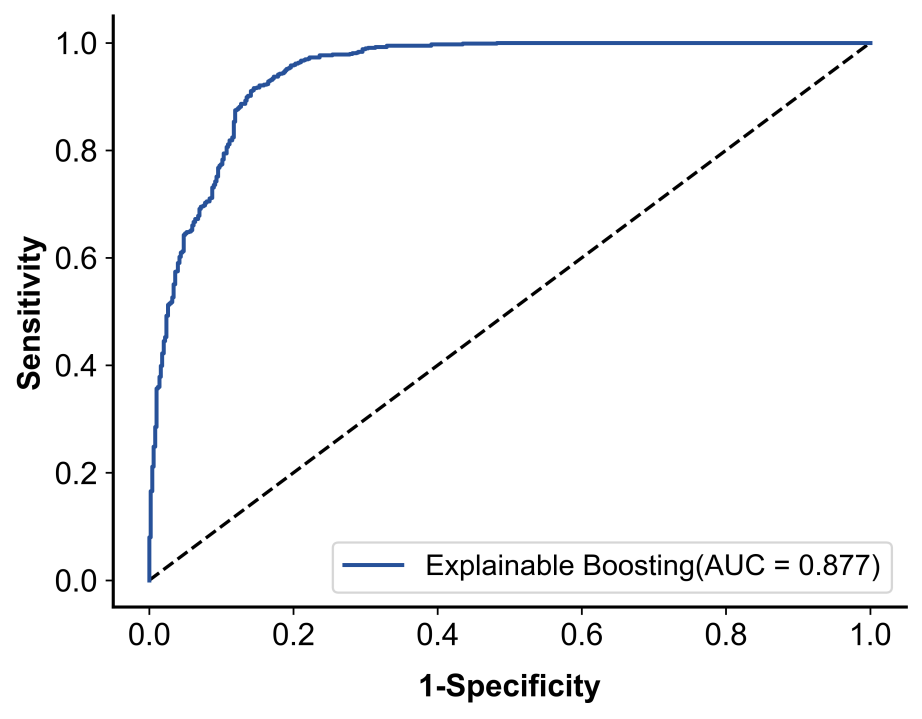


**FIGURE S3.Predicting ROC curves for the remaining malignant samples using the ExplainableBoosting model.**

| **Table s3** Performance Evaluation of Predicting Remaining Malignant Samples Using the ExplainableBoosting Model. | | | | | | |
| --- | --- | --- | --- | --- | --- | --- |
| Model | AUC(95%CI) | Accuracy | Sensitivity | Specificity | PPV | NPV |
| ExplainableBoosting | 0.876  [0.857-0.895] | 0.892  [0.876-0.901] | 0.785  [0.779-0.855] | 0.968  [0.911-0.972] | 0.875  [0.852-0.905] | 0.940  [0.895-0.963] |

Abbreviations: PPV, positive predictive value; NPV, negative predictive value; CI, confidence interval.

| **Table s4** Classification Report of Predicting Remaining Malignant Samples Using the Explainable Boosting Model. | | | | | |
| --- | --- | --- | --- | --- | --- |
| Model | Class | AUC(95%CI) | Precision | Recall | F1-score |
| ExplainableBoosting | Benign | 0.876  [0.857-0.895] | 0.94 | 0.79 | 0.86 |
|  | Maglinant |  | 0.88 | 0.97 | 0.92 |

**References**

1. Huo Z, Giger ML, Vyborny CJ, et al. Analysis of spiculation in the computerized classification of mammographic masses. Medical Physics. 1995;22(10):1569-1579.doi：[10.1118/1.597626](http://spis.hnlat.com/scholar/redirect?url=https://doi.org/10.1118/1.597626)
2. Thomas R, Piccolo F, Miller D, et al. Intrapleural fibrinolysis for the treatment of indwelling pleural catheter-related symptomatic loculations: a multicenter observational study. Chest. 2015;148(3):746-751.doi：[10.1378/chest.14-2401](http://spis.hnlat.com/scholar/redirect?url=https://doi.org/10.1378/chest.14-2401)
3. Yong LI, Pengliang WU, Zhenfei F, et al. Imaging characteristics of malignant solitary pulmonary nodule in 908 cases. J Int Med Concepts Pract. 2021;16(3):193.doi
4. Kandel P, Knipe H. Benign vs malignant pulmonary nodule. Radiopaedia.org. Accessed May 6, 2024. [doi:10.53347/rID-153664](https://doi.org/10.53347/rID-153664).
